# Impact of *Clonorchis sinensis* infection on long-term survival after curative resection for hepatocellular carcinoma: A multicenter cohort study

**DOI:** 10.1371/journal.pntd.0013441

**Published:** 2025-09-04

**Authors:** Shuang Shen, Xin Qiu, Guodong Yang, Yi Peng, Haojie Yang, Jindu Li, Jiayin Qin, Huijie Tang, Huaiyue Liang, Wenyang Zhang, Hai Huang, Ze Su, Bangde Xiang

**Affiliations:** 1 Department of Hepatobiliary Surgery, Guangxi Medical University Cancer Hospital, Nanning, China; 2 Key Laboratory of Early Prevention and Treatment for Regional High Frequency Tumor, Ministry of Education, Nanning, China; 3 Department of Gastroenterology, The First Affiliated Hospital of Guangxi Medical University, Nanning, China; 4 Department of Hepatobiliary Surgery, Changde Hospital (The First People’s Hospital of Changde), Xiangya School of Medicine, Central South University, Changde, China; 5 Oncology School, Guangxi Medical University, Nanning, China; 6 Basic Medical School, Guangxi Medical University, Nanning, China; 7 Department of Hepatobiliary Surgery, Guangxi Medical University Affiliated Wuming Hospital, Nanning, China; 8 Department of Hepatobiliary and Pancreatic Surgery, The Fifth Affiliated Hospital of Guangxi Medical University, Nanning, China; National Institute of Allergy and Infectious Diseases Division of Intramural Research, UNITED STATES OF AMERICA

## Abstract

**Background:**

Hepatocellular carcinoma (HCC) prognosis is poor in East Asia. The impact of *Clonorchis sinensis* (*C.sinensis*) infection, a known carcinogen for cholangiocarcinoma, on HCC prognosis after curative resection in co-endemic regions is unclear. This study aimed to evaluate the independent association of *C.sinensis* infection with overall survival (OS) and recurrence-free survival (RFS) after curative HCC resection.

**Methods:**

This retrospective, multicenter cohort study included 1386 patients undergoing R0 hepatectomy for HCC (2011–2021) in Guangxi, China (312 *C.sinensis*-positive, 1074 *C.sinensis*-negative). Associations were assessed using multivariable Cox regression and propensity score methods (1:1 PSM [primary], 1:3 PSM, IPTW) for multivariable adjustment for confounding. To assess robustness, additional sensitivity analyses including doubly robust estimation and E-value analysis were performed. Causal mediation analysis evaluated the role of microvascular invasion (MVI) on OS.

**Results:**

*C.sinensis* prevalence was 22.5%; median follow-up was 88 months. Significant baseline imbalances were observed. After the primary 1:1 PSM adjustment (N = 530), which achieved generally good balance (19/21 covariates SMD < 0.1), *C.sinensis* infection was significantly associated with poorer OS (adjusted Hazard Ratio [aHR], 1.55; 95% CI, 1.20–2.01; P < 0.001) and RFS (aHR, 1.63; 95% CI, 1.30–2.04; P < 0.001). The adverse OS association was robust across multivariable Cox and other propensity score sensitivity analyses (all P < 0.05). However, the RFS association was inconsistent across methods: while PSM analyses showed a significant association, this was not confirmed in multivariable Cox (P = 0.36), IPTW (P = 0.20), or doubly robust estimation (P = 0.27) analyses. After comprehensive covariate adjustment, MVI was found to significantly mediate the *C.sinensis*-OS association (Natural Indirect Effect P = 0.006), explaining approximately 12.7% (P = 0.020) of the total effect.

**Conclusion:**

Concurrent *C.sinensis* infection is an independent risk factor for reduced OS after curative HCC resection in this endemic cohort. We recommend routine preoperative screening for *C.sinensis* to improve risk stratification and guide postoperative management.

## Author summary

HCC prognosis after surgery is often poor, especially where *Clonorchis sinensis* (*C.sinensis*) liver fluke infection is common, like Guangxi, China. While *C.sinensis* causes bile duct cancer, its impact on HCC survival post-resection is unclear. We evaluated the link between *C.sinensis* infection and long-term overall survival and recurrence-free survival after curative HCC surgery.

We analyzed 1386 patients (22.5% *C.sinensis*-positive) from four Guangxi centers (2011–2021). Using statistical methods to ensure fair comparisons, we found *C.sinensis* infection was consistently linked to significantly shorter overall survival. This effect was partly explained by *C.sinensis* infection promoting cancer spread into small blood vessels, accounting for about 12.7% of the impact. However, results were inconsistent on whether *C.sinensis* infection led to earlier RFS, with different analysis methods giving different answers.

Our study identifies *C.sinensis* infection as an independent risk factor for poorer long-term survival after HCC surgery in endemic regions. Its role in cancer recurrence needs more investigation. *C.sinensis* status may be important information for predicting patient outcomes.

## 1 Introduction

Hepatocellular carcinoma (HCC) represents one of the most prevalent primary liver malignancies worldwide [1]. Its incidence and mortality rates remain persistently high, particularly in East Asian regions such as Guangxi, China, posing a significant public health challenge [2,3]. Although surgical resection is considered the principal curative treatment offering potential for long-term survival in patients with early-stage HCC, high postoperative recurrence rates and tumor progression critically limit improvements in patient survival [4,5]. Consequently, identifying risk factors influencing postoperative prognosis in patients with HCC and elucidating their underlying mechanisms is crucial for optimizing therapeutic strategies and enhancing patient outcomes.

*Clonorchis sinensis* (*C.sinensis*), commonly known as the liver fluke, is a parasitic flatworm acquired through the consumption of raw or undercooked freshwater fish and crustaceans, primarily inhabiting the intrahepatic biliary system of the host [6,7]. *C.sinensis* infection is endemic in China, especially in regions with high freshwater fish consumption like Guangxi [8]. The International Agency for Research on Cancer (IARC) has classified *C.sinensis* infection as a Group 1 biological carcinogen for cholangiocarcinoma (CCA) [9]. Its pathogenesis primarily involves chronic mechanical and chemical irritation of the biliary epithelium by the fluke and its metabolic products, leading to chronic inflammation, biliary epithelial hyperplasia, fibrosis, and eventual carcinogenesis [10,11].

Despite the established causal link between *C.sinensis* infection and CCA, its association with the development and progression of HCC remains less investigated and inconclusive. Theoretically, the pathological changes induced by *C.sinensis* infection—including chronic hepatic inflammation, cholestasis, parenchymal liver damage, and potential alterations to the immune microenvironment—could foster conditions conducive to HCC initiation or advancement [12–14]. For instance, chronic inflammation is a recognized driver of hepatocarcinogenesis [15]. Whether the persistent inflammatory response induced by *C.sinensis* infection, potentially involving infiltration by eosinophils and neutrophils, synergizes with other liver disease etiologies, such as hepatitis B virus (HBV) infection, to accelerate liver fibrosis or cirrhosis, or directly or indirectly promotes malignant biological behaviors of HCC, such as invasion and metastasis, constitutes an important scientific question warranting thorough investigation.

Despite these potential links, large-scale clinical studies evaluating the impact of *C.sinensis* infection on long-term survival outcomes following curative resection for HCC are scarce, particularly in regions like Guangxi where *C.sinensis* and HBV infections are co-endemic, limiting a comprehensive understanding of its prognostic role [15]. Leveraging the unique epidemiological landscape of the Guangxi region, this study therefore aimed to systematically evaluate the independent prognostic value of *C.sinensis* infection status for overall survival and recurrence-free survival in patients with HCC undergoing curative hepatectomy, utilizing a large retrospective dataset from four major medical centers. We hypothesized that concurrent *C.sinensis* infection is associated with poorer postoperative prognosis. To address potential confounding, rigorous statistical methods including propensity score matching and weighting were employed. Furthermore, causal mediation analysis was utilized to preliminarily explore the potential role of microvascular invasion (MVI) in the pathway linking *C.sinensis* infection to HCC prognosis. The findings are anticipated to provide critical clinical evidence regarding the role of *C.sinensis* infection within the spectrum of HCC and offer insights for risk stratification and management strategies.

## 2 Methods

### 2.1 Ethics statement

This study was approved by the Ethics Committees of Guangxi Medical University Cancer Hospital (LW2024105), the First Affiliated Hospital of Guangxi Medical University, Wuming Hospital of Guangxi Medical University and The Fifth Affiliated Hospital of Guangxi Medical University, adhering to the Declaration of Helsinki. Written consent was obtained from all hospitalized patients for the anonymous use of their medical data for analysis and research purposes.

### 2.2 Study design and patient population

This retrospective cohort study enrolled patients who underwent hepatic resection with curative intent for initially diagnosed HCC between January 2011 and May 2021. Data were aggregated from four tertiary medical centers in Guangxi, China: Guangxi Medical University Cancer Hospital, the First Affiliated Hospital of Guangxi Medical University, the Affiliated Wuming Hospital of Guangxi Medical University, and the Fifth Affiliated Hospital of Guangxi Medical University.

Inclusion criteria were: (1) age 18 years or older; (2) histologically confirmed HCC; (3) underwent curative liver resection (defined as R0 resection, achieving microscopically negative margins); and (4) received no prior anticancer therapy before surgery. Patients were excluded if they met any of the following criteria: (1) underwent palliative hepatectomy (R1 or R2 resection); (2) had concurrent malignancies other than HCC; or (3) had missing data for the primary exposure (*C.sinensis* infection status) or survival outcomes. A total of 1386 patients fulfilling these criteria were included in the final cohort. A sensitivity analysis comparing the baseline characteristics of the included and excluded cohorts was performed to assess potential selection bias (S1 Table).

### 2.3 Diagnosis of *C.sinensis* infection

A confirmed diagnosis of *C.sinensis* infection required satisfying at least one of the following criteria [16–19]: (1) preoperative imaging (magnetic resonance imaging [MRI], computed tomography [CT], microscopy, or ultrasonography) demonstrating *C.sinensis* eggs or adult flukes within the intrahepatic bile ducts; (2) a positive preoperative enzyme-linked immunosorbent assay (ELISA) serologic test for *C.sinensis*; (3) intraoperative or postoperative pathological examination revealing adult *C.sinensis* within liver or gallbladder tissue; or (4) identification of *C.sinensis* eggs via preoperative stool examination.

### 2.4 Data collection and variable definitions

Detailed baseline clinicopathological characteristics and surgery-related variables were retrieved from prospectively maintained databases at each participating center. Collected data included: (1) demographic information (age, sex); (2) preoperative laboratory parameters (serum alpha-fetoprotein [AFP], hepatitis B surface antigen [HBsAg], total bilirubin [TBil], aspartate aminotransferase [AST], alanine aminotransferase [ALT], liver disease etiology, Child-Pugh classification, Barcelona Clinic Liver Cancer [BCLC] stage; (3) complete blood counts including white blood cell [WBC], platelet [PLT], neutrophil [NEU], eosinophil [EOS], and lymphocyte [LYM] counts); and (4) postoperative pathological features (presence of cirrhosis, tumor size, tumor number, tumor encapsulation status, Edmondson-Steiner histological grade, and MVI status). For statistical modeling, certain continuous variables were categorized based on clinical relevance or established thresholds.

### 2.5 Follow-up and study endpoints

Post-hepatectomy surveillance followed a standardized protocol across all participating centers. This regimen included periodic monitoring of serum AFP levels, abdominal ultrasonography, and contrast-enhanced CT or MRI scans. Surveillance was typically performed every 2–3 months for the first two years post-surgery, and every 3–6 months thereafter. HCC recurrence was diagnosed primarily based on imaging findings demonstrating new lesions with radiological characteristics similar to the primary tumor, with or without a persistent elevation in serum AFP levels. Treatment upon recurrence varied based on clinical assessment. The primary study endpoints were overall survival (OS) and recurrence-free survival (RFS), evaluated in relation to *C.sinensis* infection status. OS was defined as the interval from the date of initial hepatectomy to the date of death from any cause or the date of the last follow-up. RFS was defined as the interval from the date of initial hepatectomy to the date of the first radiological or pathological confirmation of tumor recurrence or the date of death from any cause, or the date of the last follow-up for patients without recurrence or death.

### 2.6 Statistical analysis

Continuous variables were presented as mean ± standard deviation (SD) or median (interquartile range [IQR]), while categorical variables were summarized as frequency (n) and percentage (%). Intergroup comparisons were performed using the Student’s t-test or Mann-Whitney U test for continuous variables, and the χ^2^ test or Fisher’s exact test for categorical variables, as appropriate. Standardized mean differences (SMDs) were calculated for all baseline covariates, with an absolute SMD > 0.1 considered indicative of potential imbalance.

Survival outcomes (OS, RFS) were estimated using the Kaplan-Meier method, and survival curves were compared using the log-rank test. Univariable and multivariable Cox proportional hazards regression analyses were conducted to assess the prognostic impact of *C.sinensis* infection on survival outcomes. The multivariable models included *C.sinensi*s infection status along with all other relevant baseline demographic, laboratory, and pathological variables to adjust for potential confounding. Results were expressed as hazard ratios (HRs) (adjusted HR [aHR] for multivariable models) with their corresponding 95% confidence intervals (CIs). The proportional hazards assumption for the Cox models was assessed graphically using Schoenfeld residual plots and statistical tests; no major violations were detected. As a retrospective study, the sample size was based on all available patients meeting the inclusion criteria. A post-hoc power analysis was conducted to evaluate the statistical power for the primary endpoint.

To evaluate the robustness of the findings to potential confounding, sensitivity analyses using propensity score methods were performed. The propensity score, representing the probability of *C.sinensis* infection given baseline characteristics, was estimated using a logistic regression model that included the 8 baseline covariates identified as imbalanced: Sex, HBsAg, BCLC stage, Neu, Eos, ALB, Tumor size, and MVI. Two propensity score matching (PSM) analyses were conducted using nearest-neighbor matching without replacement based on the logit of the propensity score: (1) 1:1 matching with a caliper of 0.02 standard deviations, and (2) 1:3 matching with a caliper of 0.05 standard deviations. Additionally, inverse probability of treatment weighting (IPTW) was performed using stabilized average treatment effect (ATE) weights, truncated at the 1st and 99th percentiles. Covariate balance before and after matching/weighting was assessed across all baseline variables using SMDs and visualized with Love plots. Cox proportional hazards regression was then performed on the respective matched datasets (using weights provided by the matching procedure) or on the full cohort using the calculated IPTW weights to estimate the adjusted association between *C.sinensis* infection and survival outcomes.

To further assess the robustness of the findings, several sensitivity analyses were performed. A doubly robust estimation using an augmented inverse probability weighting (AIPW) model was conducted for the RFS outcome [20]. Additionally, to quantify the potential impact of unmeasured confounding on key findings, E-values were calculated for the primary OS association and the significant RFS association observed in the PSM analysis [21]. The E-value indicates the minimum strength of association an unmeasured confounder would need to have with both the exposure and the outcome to fully explain away the observed effect.

Furthermore, to explore potential mechanisms, a causal mediation analysis was conducted to evaluate the mediating effect of MVI on the association between *C.sinensis* infection and OS. This analysis relies on four core assumptions for identification: (1) no unmeasured exposure-outcome confounding; (2) no unmeasured mediator-outcome confounding; (3) no unmeasured exposure-mediator confounding; and (4) no mediator-outcome confounder that is itself affected by the exposure [22,23]. To better meet these assumptions, the analysis was adjusted for a comprehensive set of nine baseline covariates: age, sex, HBsAg status, liver cirrhosis, BCLC stage, ALB, AFP, tumor size, and tumor number. The natural direct effect (NDE), natural indirect effect (NIE), and the proportion mediated (PM) were estimated using a regression-based approach, which specified a logistic regression model for the binary mediator and a Cox proportional hazards model for the time-to-event outcome, with 10000 bootstrap replications for confidence intervals. A sensitivity analysis calculating the E-value for the mediator-outcome association was also performed.

Finally, two additional analyses were conducted to address specific hypotheses. First, to address the potential for bias from heterogeneous diagnostic criteria, a subgroup sensitivity analysis was performed. *C.sinensis*-positive patients were stratified into a “Confirmed Active Infection” group (diagnosed via pathology, imaging, or stool examination) and an “ELISA-Positive Only” group, and a multivariable Cox model was used to compare the OS of each subgroup to the *C.sinensis*-negative reference group. Second, to formally test for a synergistic effect with HBV, a multiplicative interaction term (*C.sinensis* status × HBsAg status) was added to the full multivariable Cox model for OS.

All statistical analyses were performed using R software, version 4.2.3 (R Foundation for Statistical Computing, Vienna, Austria). A two-tailed P value < 0.05 was considered statistically significant.

## 3 Results

### 3.1 Baseline patient characteristics

**Fig 1:**
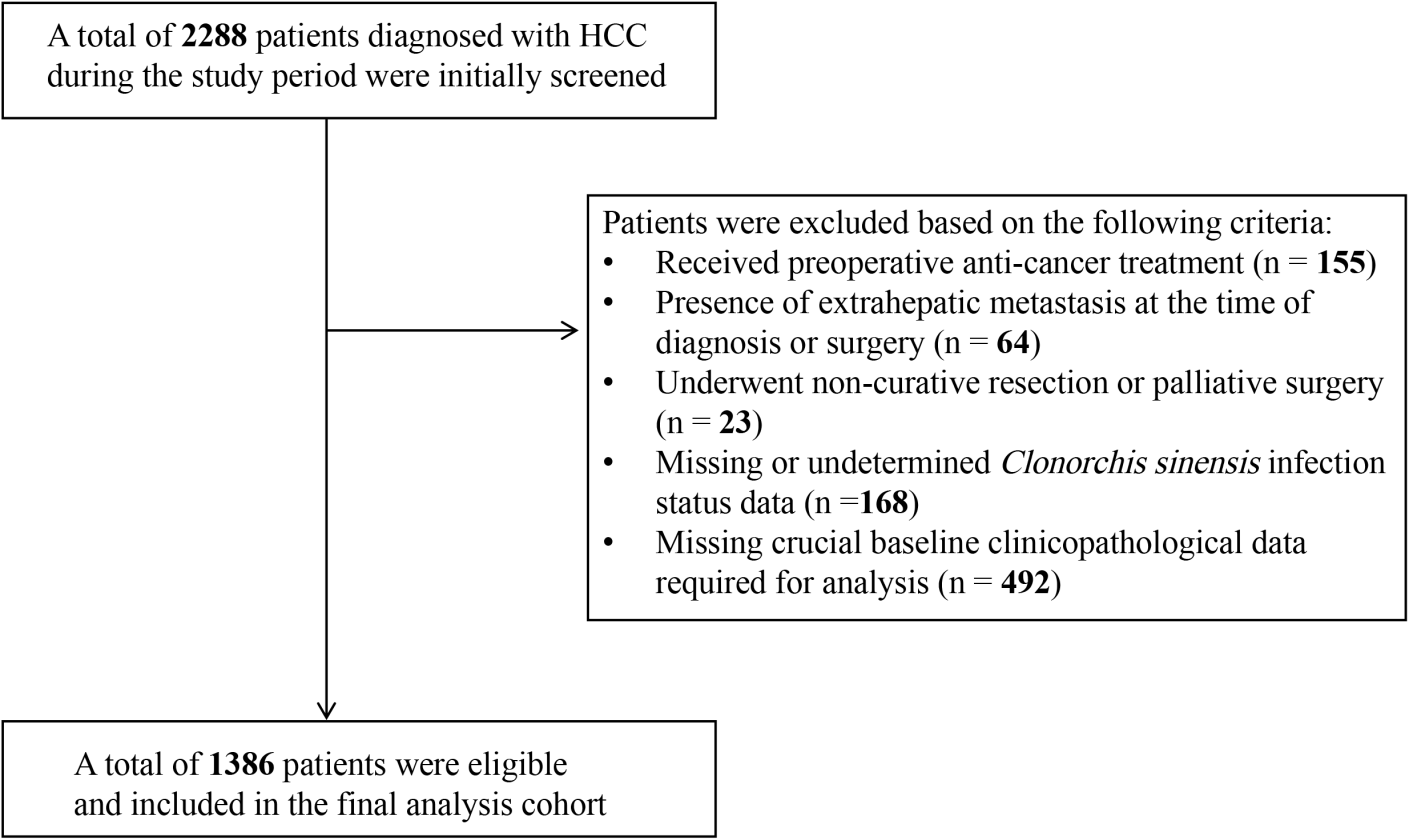
Flowchart of patient inclusion and exclusion. A total of 2288 patients diagnosed with HCC were initially screened, and 1386 eligible patients undergoing curative hepatectomy were included in the final analysis cohort.

A total of 2288 patients diagnosed with HCC were initially screened, and after applying exclusion criteria, 1386 eligible patients undergoing curative hepatectomy were included in the final analysis cohort (Fig 1). Of these, 312 (22.5%) patients were classified into the *C.sinensis*-positive group and 1074 (77.5%) into the *C.sinensis*-negative group. The median follow-up duration for the entire cohort was 88 months (95% CI: 85–91 months). Baseline clinicopathological characteristics were compared between the *C.sinensis*-positive and *C.sinensis*-negative groups (Table 1). Significant imbalances were observed for several characteristics prior to adjustment. Notably, the *C.sinensis*-positive group had a significantly higher proportion of male patients, presented more frequently with BCLC stage B-C, had a higher incidence of MVI, and exhibited higher categorized neutrophil counts, higher categorized eosinophil counts, and lower categorized albumin levels (all P < 0.001). Conversely, no statistically significant differences and minimal imbalances were observed between the groups regarding age distribution, HBsAg positivity, other routine blood counts, liver function tests, AFP levels, cirrhosis presence, major vascular invasion, or other tumor characteristics (number, size, encapsulation, grade) (Table 1). These initial baseline imbalances highlighted the potential for confounding and underscored the necessity for adjustment in subsequent survival analyses.

**Table 1:** Comparison of baseline clinicopathological characteristics between *C.sinensis*-positive and *C.sinensis*-negative patients undergoing hepatectomy for HCC.

Characteristic	*C.sinensis*(-) HCC	*C.sinensis*(+) HCC	P Value	SMD
	**n = 1074**	**n = 312**		
**Age, n(%)**			0.686	0.031
<60 years	812(75.6)	240(76.9)		
≥60 years	262(24.4)	72 (23.1)		
**Gender, n(%)**			<0.001	0.467
Female	181(16.9)	10 (3.2)		
Male	893(83.1)	302(96.8)		
**HBsAg, n(%)**				
Negative	173 (16.1)	65 (20.8)	0.062	0.122
Positive	901 (83.9)	247 (79.2)		
**BCLC stage, n(%)**				
0–A	600 (55.9)	133 (42.6)	<0.001	0.267
B–C	474 (44.1)	179 (57.4)		
**AFP, n(%)**				
<400 ng/ml	616 (57.4)	178 (57.1)	0.976	0.006
≥400 ng/ml	458 (42.6)	134 (42.9)		
**PLT, n(%)**				
≥100x10^9^/L	991 (92.3)	294 (94.2)	0.295	0.078
<100x10^9^/L	83 (7.7)	18 (5.8)		
**WBC, n(%)**				
Normal	959 (89.3)	286 (91.7)	0.265	0.081
Abnormal	115 (10.7)	26 (8.3)		
**Neu, n(%)**				
<3.82x10^9^/L	633 (58.9)	136 (43.6)	<0.001	0.311
≥3.82x10^9^/L	441 (41.1)	176 (56.4)		
**Lym, n(%)**				
<1.83x10^9^/L	592 (55.1)	170 (54.5)	0.894	0.013
≥1.83x10^9^/L	482 (44.9)	142 (45.5)		
**Eos, n(%)**				
<0.2x10^9^/L	616 (57.4)	82 (26.3)	<0.001	0.664
≥0.2x10^9^/L	458 (42.6)	230 (73.7)		
**ALB, n(%)**				
≥35g/L	921 (85.8)	221 (70.8)	<0.001	0.368
<35g/L	153 (14.2)	91 (29.2)		
**AST, n(%)**				
<40U/L	537 (50.0)	151 (48.4)	0.664	0.032
≥40U/L	537 (50.0)	161 (51.6)		
**ALT, n(%)**				
<40U/L	634 (59.0)	188 (60.3)	0.747	0.025
≥40U/L	440 (41.0)	124 (39.7)		
**TBil, n(%)**				
<17.1μmol/ml	833 (77.6)	250 (80.1)	0.374	0.063
≥17.1μmol/ml	241 (22.4)	62 (19.9)		
**Liver cirrhosis,n(%)**				
Negative	482 (44.9)	132 (42.3)	0.459	0.052
Positive	592 (55.1)	180 (57.7)		
**Macrovascular invasion,n(%)**				
Negative	902 (84.0)	253 (81.1)	0.262	0.076
Positive	172 (16.0)	59 (18.9)		
**Number of tumors, n(%)**				
Solitary	725 (67.5)	204 (65.4)	0.527	0.045
Multiple	349 (32.5)	108 (34.6)		
**Tumor size, n(%)**				
<5 cm	622 (57.9)	165 (52.9)	0.13	0.101
≥5 cm	452 (42.1)	147 (47.1)		
**Capsule of tumor, n(%)**				
Complete	339 (31.6)	107 (34.3)	0.401	0.058
Incomplete	735 (68.4)	205 (65.7)		
**Edmonson grade, n(%)**				
I-II	530 (49.3)	154 (49.4)	1	<0.001
III-IV	544 (50.7)	158 (50.6)		
**MVI, n(%)**				
Negative	629 (58.6)	140 (44.9)	<0.001	0.277
Positive	445 (41.4)	172 (55.1)		

**Abbreviations:** *C.s*inensis**: *Clonorchis sinensis*, (+) indicates infection, (-) indicates no infection; HCC: Hepatocellular Carcinoma; n: Number; %: Percentage; P Value: Probability value (indicates statistical significance); SMD: Standardized Mean Difference (effect size); HBsAg: Hepatitis B surface antigen; BCLC: Barcelona Clinic Liver Cancer staging; AFP: Alpha-fetoprotein; PLT: Platelet count; WBC: White Blood Cell count; Neu: Neutrophil count; Lym: Lymphocyte count; Eos: Eosinophil count; ALB: Albumin; AST: Aspartate aminotransferase; ALT: Alanine aminotransferase; TBil: Total Bilirubin; MVI: Microvascular Invasion; Edmonson grade: Edmonson-Steiner histological grade for HCC differentiation.

**Categorization Thresholds:** WBC was categorized as ‘Abnormal’ if outside the standard range of (3.5–9.5)×10⁹/L. PLT was categorized as ‘<100 × 10⁹/L’. Neu was categorized based on a cutoff of 3.82 × 10⁹/L. Lym was categorized based on a cutoff of 1.83 × 10⁹/L. Eos was categorized based on a cutoff of 0.2 × 10⁹/L. ALB was categorized based on a cutoff of 35g/L. AST and ALT were categorized based on a cutoff of 40U/L. TBil was categorized based on a cutoff of 17.1μmol/L. AFP was categorized based on a cutoff of 400ng/ml. Tumor size was categorized based on a cutoff of 5 cm. These thresholds are based on standard clinical reference ranges and established literature.

### 3.2 Unadjusted survival analysis

**Fig 2:**
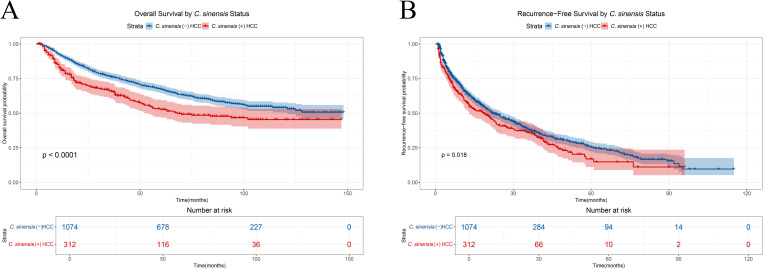
A. Unadjusted Overall Survival (OS) by *C.sinensis* Infection Status. B. Unadjusted Recurrence-Free Survival (RFS) by *C.sinensis* Infection Status.

In unadjusted analyses, Kaplan-Meier survival curves revealed significant differences between the *C.sinensis*-positive and *C.sinensis*-negative groups for both OS and RFS. For OS, *C.sinensis*-positive patients experienced significantly poorer survival (Log-rank P < 0.001; Fig 2A), with a median OS of 68 months (95% CI: 52–Not Reached [NR]; n = 312, 135 events) compared to NR (95% CI: 121–NR) for *C.sinensis*-negative patients (n = 1074, 424 events). Similarly, *C.sinensis*-positive patients demonstrated significantly shorter RFS (Log-rank P = 0.02; Fig 2B), with a median RFS of 18.7 months (95% CI: 13.0–23.3; n = 312, 186 events/recurrences) compared to 22.0 months (95% CI: 19.8–26.3) in the *C.sinensis*-negative group (n = 1074, 611 events/recurrences).

### 3.3 Multivariable cox regression analysis

To evaluate the independent prognostic significance of *C.sinensis* infection after adjusting for baseline confounding factors, multivariable Cox proportional hazards regression models were employed. In the multivariable model for OS (N = 1386, 559 events; model P < 0.001), *C.sinensis* infection remained an independent predictor of poorer survival (aHR = 1.26, 95% CI: 1.03–1.55, P = 0.024). Other significant independent predictors for worse OS included BCLC stage B-C vs 0-A (aHR = 2.20, P < 0.001), higher platelet category (aHR = 1.56, P = 0.003), higher neutrophil category (aHR = 1.22, P = 0.022), and presence of MVI (aHR = 1.33, P = 0.001), while higher lymphocyte category was associated with better OS (aHR = 0.80, P = 0.011) (Table 2). However, for RFS (N = 1386, 797 events; model P < 0.001), *C.sinensis* infection was not found to be an independent predictor after adjustment in the multivariable model (aHR = 1.08, 95% CI: 0.91–1.28, P = 0.359). In this RFS model, BCLC stage (aHR = 1.81, P < 0.001) and MVI (aHR = 1.37, P < 0.001) were independent risk factors for worse RFS, whereas higher lymphocyte category remained protective (aHR = 0.86, P = 0.034) (Table 3).

**Table 2:** Univariable and multivariable cox regression analysis of factors associated with overall survival in hepatocellular carcinoma patients.

	HR comparison	HR (univariable)	HR (multivariable)
***C.sinensis***	Positive vs. Negative	1.51(1.24-1.83, p < 0.001)	1.26(1.03-1.55, p = 0.024)
**Age**	≥60 vs. < 60 years	1.07(0.89-1.30, p = 0.457)	
**Gender**	Male vs. Female	1.39(1.07-1.81, p = 0.013)	1.27(0.97-1.65, p = 0.084)
**HBsAg**	Positive vs. Negative	1.00(0.80-1.25, p = 0.994)	
**BCLC stage**	B-C vs. 0-A	2.35(1.98-2.79, p < 0.001)	2.20(1.84-2.61, p < 0.001)
**WBC**	Abnormal vs. Normal	1.14(0.88-1.49, p = 0.316)	
**PLT**	<100 vs. ≥ 100x10^9^/L	1.41(1.06-1.89, p = 0.018)	1.56(1.16-2.10, p = 0.003)
**Neu**	≥3.82 vs. < 3.82x10^9^/L	1.31(1.11-1.55, p = 0.001)	1.22(1.03-1.45, p = 0.022)
**Lym**	≥1.83 vs. < 1.83x10^9^/L	0.79(0.66-0.93, p = 0.005)	0.80(0.68-0.95, p = 0.011)
**Eos**	≥0.2 vs. < 0.2x10^9^/L	1.16(0.98-1.36, p = 0.086)	
**ALB**	<35 vs. ≥ 35g/L	1.25(1.00-1.56, p = 0.046)	1.14(0.91-1.43, p = 0.240)
**AST**	≥40 vs. < 40U/L	1.06(0.90-1.25, p = 0.485)	
**ALT**	≥40 vs. < 40U/L	0.99(0.83-1.17, p = 0.877)	
**TBil**	≥17.1 vs. < 17.1μmol/ml	1.03(0.84-1.26, p = 0.774)	
**AFP**	≥400 vs. < 400ng/ml	1.05(0.89-1.24, p = 0.544)	
**Liver cirrhosis**	Positive vs. Negative	1.05(0.89-1.24, p = 0.557)	
**Macrovascular invasion**	Positive vs. Negative	1.07(0.86-1.33, p = 0.545)	
**Number of tumors**	Multiple vs. Solitary	0.97(0.81-1.16, p = 0.753)	
**Tumor size**	≥5 vs. < 5 cm	1.06(0.90-1.26, p = 0.483)	
**Capsule of tumor**	Incomplete vs. Complete	1.01(0.84-1.20, p = 0.939)	
**Edmonson grade**	III-IV vs. I-II	1.00(0.85-1.18, p = 0.971)	
**MVI**	Positive vs. Negative	1.52(1.29-1.80, p < 0.001)	1.33(1.13-1.58, p = 0.001)

**Abbreviations:** *C.sinensis*: *Clonorchis sinensis*; HR: Hazard Ratio; CI: Confidence Interval; p: p-value; HBsAg: Hepatitis B surface antigen; BCLC: Barcelona Clinic Liver Cancer staging; WBC: White Blood Cell count; PLT: Platelet count; Neu: Neutrophil count; Lym: Lymphocyte count; Eos: Eosinophil count; ALB: Albumin; AST: Aspartate aminotransferase; ALT: Alanine aminotransferase; TBil: Total Bilirubin; AFP: Alpha-fetoprotein; MVI: Microvascular Invasion.

**Table 3:** Univariable and multivariable cox regression analysis of factors associated with recurrence-free survival in hepatocellular carcinoma patients.

Variables	HR comparison	HR (univariable)	HR (multivariable)
***C.sinensis***	Positive vs. Negative	1.22(1.03-1.44, p = 0.019)	1.08(0.91-1.28, p = 0.359)
**Age**	≥60 vs. < 60 years	0.88(0.75-1.04, p = 0.136)	
**Gender**	Male vs. Female	1.24(1.01-1.53, p = 0.044)	1.13(0.91-1.39, p = 0.278)
**HBsAg**	Positive vs. Negative	1.16(0.96-1.40, p = 0.117)	
**BCLC stage**	B-C vs. 0-A	1.91(1.66-2.20, p < 0.001)	1.81(1.57-2.09, p < 0.001)
**WBC**	Abnormal vs. Normal	1.06(0.84-1.34, p = 0.618)	
**PLT**	<100 vs. ≥ 100x10^9^/L	0.96(0.74-1.25, p = 0.743)	
**Neu**	≥3.82 vs. < 3.82x10^9^/L	1.11(0.96-1.27, p = 0.154)	
**Lym**	≥1.83 vs. < 1.83x10^9^/L	0.85(0.74-0.98, p = 0.026)	0.86(0.75-0.99, p = 0.034)
**Eos**	≥0.2 vs. < 0.2x10^9^/L	1.11(0.96-1.27, p = 0.153)	
**ALB**	<35 vs. ≥ 35g/L	1.13(0.94-1.37, p = 0.187)	
**AST**	≥40 vs. < 40U/L	1.03(0.90-1.19, p = 0.654)	
**ALT**	≥40 vs. < 40U/L	0.98(0.85-1.13, p = 0.766)	
**TBil**	≥17.1 vs. < 17.1μmol/ml	0.93(0.78-1.10, p = 0.407)	
**AFP**	≥400 vs. < 400ng/ml	1.07(0.93-1.23, p = 0.354)	
**Liver cirrhosis**	Positive vs. Negative	1.07(0.93-1.23, p = 0.341)	
**Macrovascular invasion**	Positive vs. Negative	0.89(0.74-1.07, p = 0.214)	
**Number of tumors**	Multiple vs. Solitary	0.98(0.85-1.14, p = 0.808)	
**Tumor size**	≥5 vs. < 5 cm	1.11(0.96-1.27, p = 0.160)	
**Capsule of tumor**	Incomplete vs. Complete	1.07(0.92-1.25, p = 0.354)	
**Edmonson grade**	III-IV vs. I-II	0.99(0.86-1.14, p = 0.894)	
**MVI**	Positive vs. Negative	1.49(1.29-1.71, p < 0.001)	1.37(1.19-1.58, p < 0.001)

**Abbreviations:** *C.sinensis*: *Clonorchis sinensis*; HR: Hazard Ratio; CI: Confidence Interval; p: p-value; HBsAg: Hepatitis B surface antigen; BCLC: Barcelona Clinic Liver Cancer staging; WBC: White Blood Cell count; PLT: Platelet count; Neu: Neutrophil count; Lym: Lymphocyte count; Eos: Eosinophil count; ALB: Albumin; AST: Aspartate aminotransferase; ALT: Alanine aminotransferase; TBil: Total Bilirubin; AFP: Alpha-fetoprotein; MVI: Microvascular Invasion.

### 3.4 Propensity score sensitivity analyses

**Fig 3:**
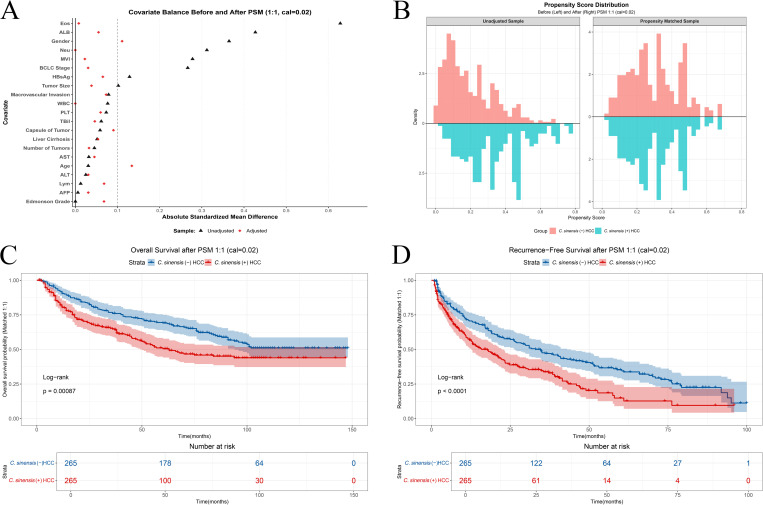
Propensity Score Matching (PSM) 1:1 (Caliper 0.02) Analysis Results. (A) Love plot showing the absolute standardized mean difference (SMD) for baseline covariates before (Unadjusted, triangles) and after (Adjusted, diamonds) matching. The dashed vertical line indicates the SMD threshold of 0.1. (B) Distribution of propensity scores before (Unadjusted Sample, left) and after (Propensity Matched Sample, right) matching, stratified by *C.sinensis* infection status (*C.sinensis*-: Reddish, *C.sinensis* + : Tealish). (C) Kaplan-Meier curve comparing overall survival (OS) between *C.sinensis*-positive and *C.sinensis*-negative groups in the 1:1 matched cohort. (D) Kaplan-Meier curve comparing recurrence-free survival (RFS) between *C.sinensis*-positive and *C.sinensis*-negative groups in the 1:1 matched cohort. P-values in (C) and (D) are from the log-rank test.

To control for baseline confounding using an alternative approach, we primarily employed PSM using a 1:1 nearest-neighbor algorithm with a caliper of 0.02. The propensity score model included the 8 baseline covariates initially imbalanced: Sex, HBsAg, BCLC stage, Neu, Eos, ALB, Tumor size, and MVI. This procedure yielded 265 matched pairs (N = 530). Visualization of the propensity score distributions confirmed improved overlap between the *C.sinensis*-positive and *C.sinensis*-negative groups after 1:1 matching compared to the unadjusted sample (Fig 3B). Assessment of covariate balance after 1:1 matching demonstrated substantial improvement across all baseline covariates; SMDs for 19 out of 21 variables were reduced below the 0.1 threshold, although minor imbalances persisted for Age and Gender (Fig 3A and S2 Table). In this primary propensity score adjusted analysis, *C.sinensis* infection was significantly associated with poorer OS (aHR = 1.55, 95% CI: 1.20–2.01, P < 0.001) and also with poorer RFS (aHR = 1.63, 95% CI: 1.30–2.04, P < 0.001) (Fig 3C and 3D).

To assess the robustness of these findings, several sensitivity analyses were conducted. First, a less stringent 1:3 nearest-neighbor PSM (caliper 0.05) resulted in a larger cohort (n = 900). While balance was improved compared to the unadjusted state, it was less optimal than 1:1 matching (S3 Table). In this 1:3 matched cohort, *C.sinensis* infection remained significantly associated with poorer OS (Log-rank P = 0.001; S1A Fig) and was also significantly associated with poorer RFS (Log-rank P = 0.014; S1B Fig). Second, IPTW analysis using trimmed stabilized ATE weights in the full cohort confirmed a significant association with reduced OS (aHR = 1.36, P < 0.001; S1C Fig) but no significant association with RFS (aHR = 1.13, P = 0.197; S1D Fig). However, the IPTW results require cautious interpretation due to poor balance observed for Gender (S4 Table).

In summary, sensitivity analyses consistently supported the finding that *C.sinensis* infection is robustly associated with poorer OS after adjusting for observed confounders. In contrast, the association with RFS, while found to be significant in both 1:1 and 1:3 PSM analyses, was not significant in the conventional multivariable Cox regression or the IPTW analysis (which had balance limitations). This suggests the RFS finding may be more sensitive to the specific analytical method and the quality of covariate balance achieved.

To further adjudicate the inconsistent RFS findings, AIPW was performed on the full cohort. The result showed no significant association between *C.sinensis* infection and RFS (aHR = 1.11, 95% CI: 0.92–1.35, P = 0.27), supporting the conclusions from the multivariable Cox and IPTW models. For the significant RFS finding in the 1:1 PSM analysis (aHR = 1.63), the calculated E-value was 2.15, indicating that an unmeasured confounder would need to be associated with both *C.sinensis* infection and recurrence with a risk ratio of at least 2.15 each, above and beyond the measured covariates, to fully explain away this effect (S5 Table).

### 3.5 Mediation analysis: Role of MVI in OS

**Fig 4:**
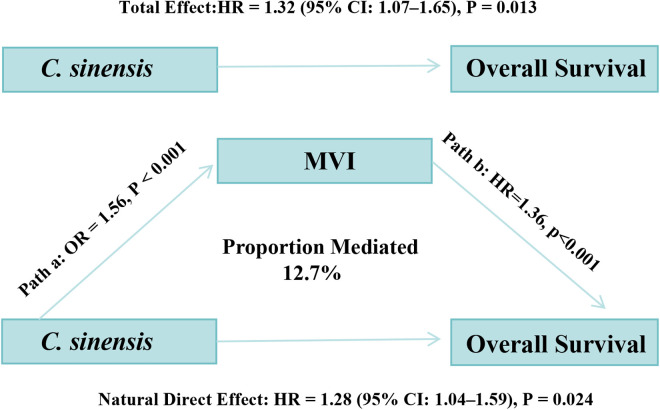
Path diagram illustrating the mediation analysis of the effect of *C.sinensis* infection on overall survival (OS) through microvascular invasion (MVI), The analysis was adjusted for a comprehensive set of nine baseline covariates: age, sex, HBsAg status, liver cirrhosis, BCLC stage, albumin, AFP, tumor size, and tumor number.

To explore potential pathways linking *C.sinensis* infection to OS, a causal mediation analysis with comprehensive covariate adjustment evaluated MVI as a potential mediator. The analysis confirmed a significant total effect of *C.sinensis* infection on OS (HR = 1.32, 95% CI: 1.07–1.65, P = 0.013). Both the NDE (HR = 1.28, 95% CI: 1.04–1.59, P = 0.024) and the NIE through MVI (HR = 1.03, 95% CI: 1.01–1.07, P = 0.006) were statistically significant. MVI was estimated to mediate 12.7% (95% CI: 2.9%–45.9%, P = 0.020) of the total effect of *C.sinensis* on OS (Fig 4). A sensitivity analysis for the mediator-outcome link yielded an E-value of 1.78 for the observed HR of 1.36, suggesting a moderate degree of robustness to unmeasured confounding (S5 Table). These findings suggest that while *C.sinensis* infection primarily exerts a direct adverse effect on OS, MVI acts as a statistically significant partial mediator in this relationship.

### 3.6 Interaction analysis with HBV

To investigate a potential synergistic effect between *C.sinensis* and HBV, a formal interaction analysis was conducted. The analysis revealed no statistically significant interaction between *C.sinensis* infection and HBsAg status on OS (P for interaction = 0.174) (S6 Table). This suggests that the prognostic impact of *C.sinensis* infection does not significantly differ between HBsAg-positive and HBsAg-negative patients.

### 3.7 Subgroup sensitivity analysis by diagnostic method

To investigate whether the prognostic association was driven by specific diagnostic methods, a subgroup analysis was performed. Among the 312 *C.sinensis*-positive patients, 271 (86.9%) were classified into the “Confirmed Active Infection” group, and 41 (13.1%) were in the “ELISA-Positive Only” group. Baseline characteristics for these subgroups are detailed in S7 Table.

In the multivariable Cox model for OS, the “Confirmed Active Infection” group remained significantly associated with poorer survival compared to the *C.sinensis*-negative group (aHR = 1.25, 95% CI: 1.00–1.56, P = 0.045). In contrast, the “ELISA-Positive Only” group showed no significant association with OS (aHR = 1.15, 95% CI: 0.67–1.99, P = 0.612). For RFS, neither subgroup showed a significant association after multivariable adjustment (S7 Table). These results demonstrate that the primary finding of an adverse OS association is robust and driven by patients with definitive evidence of active infection.

## 4 Discussion

This large-scale, retrospective cohort study, encompassing 1386 patients undergoing curative hepatectomy for HCC in the Guangxi region of China, establishes *C.sinensis* infection as a robust and independent risk factor for poorer long-term OS. The 22.5% prevalence of *C.sinensis* infection within this cohort highlights it as a non-negligible condition among patients with HCC in this endemic region. Patients in the *C.sinensis*-positive group were more likely to be male, present at a more advanced BCLC stage, have MVI, exhibit elevated neutrophil and eosinophil counts, and have lower albumin levels compared to the *C.sinensis*-negative group.

The central finding of this study is the independent and robust association between *C.sinensis* infection and reduced OS following curative resection for HCC. After comprehensive adjustment for numerous potential confounders using multivariable Cox regression (aHR = 1.26, 95% CI: 1.03–1.55, P = 0.024), this association remained significant and was consistently validated across multiple propensity score-based sensitivity analyses. A key concern was the heterogeneity of diagnostic criteria. However, our subgroup analysis demonstrated that this adverse association was driven entirely by the “Confirmed Active Infection” group (aHR = 1.25, P = 0.045), an effect size nearly identical to our primary analysis, while the “ELISA-Positive Only” group showed no significant risk. This provides strong evidence that our main conclusion is not an artifact of misclassification from serology but is instead linked to active parasitic infection. Given the high prevalence of HBV in our cohort, we formally tested for a synergistic effect with *C.sinensis* infection. Our interaction analysis showed no significant statistical interaction (P = 0.174), suggesting *C.sinensis* acts as an independent risk factor for poorer OS regardless of HBV status. This finding is consistent with previous research from our group [24], which also concluded that HCC patients with *C.sinensis* infection experience a poor prognosis after hepatectomy, irrespective of HBV co-infection.

In contrast to the consistent findings for OS, the association between *C.sinensis* infection and RFS was method-dependent, a finding that offers insight into the nature of the association. While stricter PSM methods (1:1 PSM) identified a significant link to poorer RFS, this was not confirmed in analyses of the full cohort using multivariable regression (aHR = 1.08, P = 0.359) or doubly robust estimation (aHR = 1.11, P = 0.27). This discrepancy should not be viewed as a contradiction, but rather as a reflection of different methods answering different scientific questions. PSM creates a highly comparable, quasi-randomized subgroup by sacrificing sample size and generalizability; in this ideal subgroup, a significant association with earlier recurrence was detected. Conversely, regression-based methods utilize the entire, more heterogeneous cohort, preserving statistical power but relying on model assumptions. The null finding in these latter analyses suggests that while the effect on RFS may be present in specific patient subsets, it is likely modest and not consistently detectable across the broader patient population.

Our causal mediation analysis, now strengthened by comprehensive adjustment for nine key confounders, provided an important mechanistic clue by identifying MVI as a statistically significant partial mediator of the *C.sinensis* effect on OS. The analysis confirmed that MVI mediates approximately 12.7% of the total effect. This suggests *C.sinensis* infection may promote MVI formation. Indeed, recent evidence supports this finding; studies from our region have shown that *C.sinensis* infection enhances angiogenesis in HCC, evidenced by increased microvessel density (MVD) and upregulation of key angiogenic factors like VEGF in tumor tissues [25]. This pro-angiogenic environment could directly facilitate microvascular invasion.

However, the finding that the majority (approximately 87%) of the *C.sinensis* effect on OS remains direct indicates that other mechanisms independent of MVI play a predominant role. Several pathways supported by recent literature are plausible contributors. First, chronic inflammation and immune microenvironment remodeling are central to *C.sinensis* pathogenesis. The parasite induces a chronic inflammatory state which can promote tumor progression by altering immune surveillance. Recent work has shown that *C.sinensis*-infected HCC exhibits an immunosuppressive tumor microenvironment characterized by an accumulation of resting dendritic cells and impaired T-cell activity [13]. Second, *C.sinensis* infection may promote cancer stemness. A study in a similar Guangxi cohort demonstrated that the infection is associated with increased expression of HCC stem cell markers CK19 and EpCAM, which are linked to aggressive tumor biology and poor prognosis [26]. Third, emerging evidence points to parasite-induced metabolic reprogramming. *C.sinensis* and its excretory/secretory products can alter host cell metabolism, including arginine biosynthesis and glucose utilization pathways, which can fuel tumor growth while impairing anti-tumor immunity [27]. These pathways likely constitute a significant portion of the ‘direct effect’ observed in our study and represent critical directions for future mechanistic research.

This study possesses several strengths, including its large sample size and the use of advanced statistical methods. Nevertheless, certain limitations should be acknowledged. First, the retrospective design is inherently susceptible to selection bias and unmeasured confounding. We excluded 492 patients due to missing primary exposure or outcome data, which could introduce bias. However, our comparative analysis (S1 Table) revealed a complex, non-unidirectional pattern of differences between the included and excluded cohorts; the excluded group presented with a mix of both better (e.g., earlier BCLC stage) and worse (e.g., larger tumors) prognostic factors. This finding argues against a simple, systematic selection bias (e.g., excluding only the sickest patients) and suggests the net direction of any potential bias is difficult to ascertain. To quantify the potential impact of unmeasured confounding, we calculated an E-value for the primary OS association (aHR = 1.26), which was 1.63. While this suggests a degree of robustness, we urge cautious interpretation. Furthermore, while our primary OS finding was significant, a post-hoc power analysis indicated a statistical power of 67.9% for our primary endpoint, which is below the conventional 80% threshold. This limited power should be considered when interpreting our findings. On one hand, it may have limited our ability to consistently detect a more modest association for RFS across all analytical methods, potentially explaining the observed inconsistency in those results. On the other hand, the fact that a statistically significant effect was detected for our primary endpoint (OS) despite this limited power reinforces the robustness of that key finding, as a less pronounced effect might have been missed. Additional limitations include the lack of data on infection severity or anti-parasitic treatment history, potential inter-observer variability in MVI assessment, and the fact that our findings from a high HBV prevalence region may not be directly generalizable to other populations [28].

## 5 Clinical implications and future directions

The findings hold significant clinical importance. Preoperative assessment of *C.sinensis* status in endemic regions might aid risk stratification for HCC patients undergoing resection, potentially indicating a need for closer surveillance in co-infected individuals. While current evidence is lacking, the question arises whether active anti-parasitic treatment could improve long-term survival in co-infected HCC patients, a hypothesis meriting investigation through future prospective trials. These results may also prompt reconsideration of *C.sinensis* infection’s role in the broader spectrum of liver diseases. Future research should prioritize prospective cohort studies with detailed *C.sinensis* infection data, mechanistic studies using multi-omics and immunological approaches to elucidate biological pathways, multi-regional collaborations to assess generalizability, and potentially, well-designed interventional trials evaluating anti-*C.sinensis* treatment in the context of HCC prognosis.

In conclusion, this large, multicenter cohort study demonstrates that *C.sinensis* infection is an independent and robust risk factor for reduced OS in patients undergoing curative resection for HCC in the Guangxi region of China. These findings have significant clinical implications for patient management in endemic areas. We recommend that routine preoperative screening for *C.sinensis* infection be considered for incorporation into the standard clinical workup for all patients diagnosed with HCC. Identifying co-infected patients would allow for more accurate prognostic stratification and could trigger more intensive postoperative surveillance protocols. Furthermore, our findings raise the critical question of whether anti-parasitic treatment could improve long-term outcomes in these patients, providing a strong rationale for future prospective, randomized controlled trials.

## Supporting information

S1 Data Minimal dataset. The fully de-identified minimal dataset necessary to replicate the study’s findings.
(CSV)

S1 Text Codebook. Data dictionary. A comprehensive data dictionary explaining the variables and coding used in the S1 Data file.
(DOCX)

S1 Fig Kaplan-Meier curves for overall survival (OS) and recurrence-free survival (RFS) from sensitivity analyses Kaplan-Meier curves comparing outcomes between *C.sinensis*-positive and *C.sinensis*-negative groups after adjustment using different propensity score methods. (A) OS after 1:3 PSM. (B) RFS after 1:3 PSM. (C) OS after IPTW with trimmed weights. (D) RFS after IPTW with trimmed weights.
(TIF)

S1 Table Comparison of baseline characteristics between included and excluded cohorts. (XLS)

S2 Table Covariate balance after 1:1 propensity score matching. (XLS)

S3 Table Covariate balance after 1:3 propensity score matching. (XLS)

S4 Table Covariate balance after inverse probability of treatment weighting. (XLS)

S5 Table Sensitivity analyses: Doubly robust estimation and E-value analysis. (XLS)

S6 Table Multivariable cox regression analysis of the interaction between *C.sinensis* and HBsAg status on overall survival. (XLS)

S7 Table Baseline characteristics and survival analysis by *C.sinensis* diagnostic subgroup. (XLS)
