# Supplementary material for: Impact of Clonorchis sinensis infection on long-term survival after curative resection for hepatocellular carcinoma: A multicenter cohort study
Source: PLoS Negl Trop Dis. 2025 Sep 4;19(9):e0013441. doi: 10.1371/journal.pntd.0013441 (PMC12419606; doi:10.1371/journal.pntd.0013441)
Supplement: S1 Text — Data dictionary. A comprehensive data dictionary explaining the variables and coding used in the S1 Data file. (DOCX) [file pntd.0013441.s002.docx]

**S1 Codebook. Data dictionary. A comprehensive data dictionary explaining the variables and coding used in the S1 Data file**

| **Variable Name** | **Description** | **Coding** |
| --- | --- | --- |
| ID | Unique anonymous identifier for each patient | N/A |
| *Clonorchis_sinensis* | *Clonorchis sinensis* infection status | 1 = Positive; 0 = Negative |
| Age | Age group | 1 = ≥60 years; 0 = <60 years |
| Gender | Gender | 1 = Male; 0 = Female |
| HBsAg | Hepatitis B surface antigen status | 1 = Positive; 0 = Negative |
| BCLC_group | Barcelona Clinic Liver Cancer stage | 1 = Stage 0-A; 2 = Stage B-C |
| WBC_group | White Blood Cell count group | 1 = Abnormal; 0 = Normal |
| PLT_group | Platelet count group | 1 = <100x10⁹/L; 0 = ≥100x10⁹/L |
| Neu_group | Neutrophil count group | 1 = ≥3.82x10⁹/L; 0 = <3.82x10⁹/L |
| Lym_group | Lymphocyte count group | 1 = ≥1.83x10⁹/L; 0 = <1.83x10⁹/L |
| Eos_group | Eosinophil count group | 1 = ≥0.2x10⁹/L; 0 = <0.2x10⁹/L |
| ALB_group | Albumin level group | 1 = <35g/L; 0 = ≥35g/L |
| AST_group | Aspartate aminotransferase group | 1 = ≥40U/L; 0 = <40U/L |
| ALT_group | Alanine aminotransferase group | 1 = ≥40U/L; 0 = <40U/L |
| TBil_group | Total Bilirubin group | 1 = ≥17.1μmol/L; 0 = <17.1μmol/L |
| AFP_group | Alpha-fetoprotein group | 2 = ≥400ng/ml; 1 = <400ng/ml |
| Liver_cirrhosis | Presence of liver cirrhosis | 1 = Positive; 0 = Negative |
| cancer_embolus | Presence of macrovascular invasion | 1 = Positive; 0 = Negative |
| Number_of_tumors | Number of tumors | 2 = Multiple; 1 = Solitary |
| Tumor_size | Largest tumor diameter | 1 = ≥5cm; 0 = <5cm |
| Capsule_of_tumor | Tumor capsule status | 1 = Incomplete; 0 = Complete |
| Edmonson_group | Edmonson-Steiner histological grade | 2 = Grade III-IV; 1 = Grade I-II |
| MVI | Microvascular invasion status | 1 = Positive; 0 = Negative |
| RFS | Recurrence-free survival time | In months |
| RFSstat | Recurrence-free survival status | 1 = Event (recurrence or death); 0 = Censored |
| OS | Overall survival time | In months |
| OSstat | Overall survival status | 1 = Event (death); 0 = Censored |
| Diag_Imaging | Diagnosis by imaging | 1 = Yes; 0 = No |
| Diag_ELISA | Diagnosis by ELISA | 1 = Yes; 0 = No |
| Diag_Pathology | Diagnosis by pathology | 1 = Yes; 0 = No |
| Diag_Stool | Diagnosis by stool examination | 1 = Yes; 0 = No |
