# Supplementary figures and images for: Impact of Clonorchis sinensis infection on long-term survival after curative resection for hepatocellular carcinoma: A multicenter cohort study
Source: PLoS Negl Trop Dis. 2025 Sep 4;19(9):e0013441. doi: 10.1371/journal.pntd.0013441 (PMC12419606; doi:10.1371/journal.pntd.0013441)

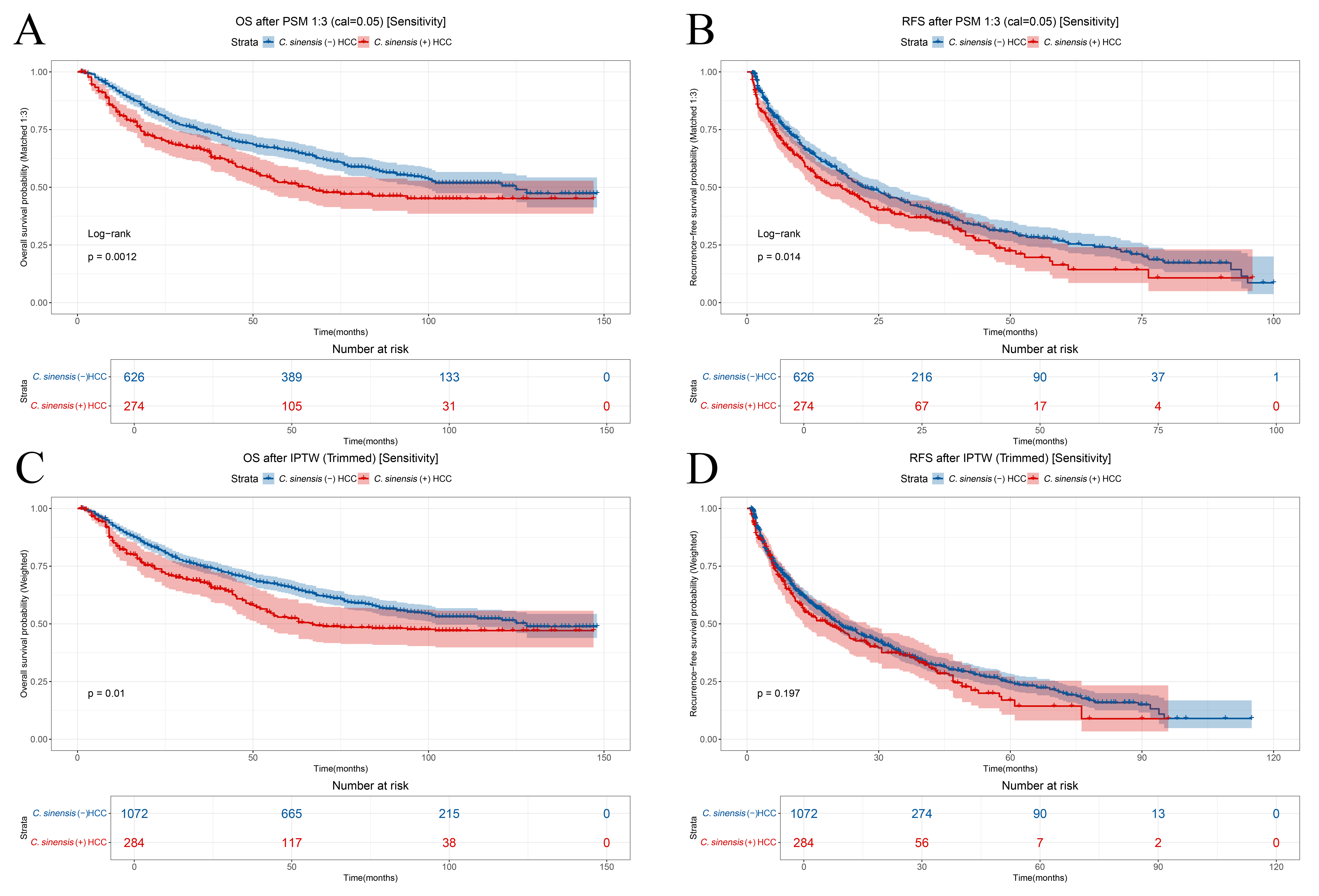

Supplement: S1 Fig — (A) OS after 1:3 PSM. (B) RFS after 1:3 PSM. (C) OS after IPTW with trimmed weights. (D) RFS after IPTW with trimmed weights. (TIF) [file pntd.0013441.s003.tif]
